# An Evening with Beau Nash and Juliana Popjoy

**Published:** 1988-02

**Authors:** Jeremy. J. Mann


### An Evening with Beau Nash and Juliana Popjoy

The ghost of Juliana Popjoy has been reported as being seen in the large elegant upstairs drawing room, into which early arrivals are shown to have drinks before dinner. Popjoys restaurant now occupies the house of Beau Nash's mistress. They would both be suitably impressed with the style panache and excellence of the fayre at one of the best restaurants in the west.

Beau Nash himself looks on from a large subtly lit portrait on the wall of the tastefully decorated downstairs dining room, kept at a comfortable temperature by the open fire in the grate.

Choose from the large set priced menu, any one of the many starters main courses and desserts all for an inclusive £18–50. We found choosing very difficult as everything on the menu is so tempting: Feuillete with hot ratatouille and Brie; smoked monkfish; rabbit and pigeon terrine; cauliflower soup, eventually I went for the timbale of chicken livers on a wood mushroom and herb jelly, served with a warm brioche. My wife enjoyed the mussels and artichoke gratin immensely, subtle and exciting.

To follow the decision was even more difficult—the fresh fish of the day, salmon and brill; beef fillet in pink peppercorn sauce; oxtail braised in red wine; roast Guinea fowl with garlic and bacon in a Port wine sauce. I settled for the Dutch loin of veal with tartlette of calves kidneys in a rosemary sauce, this was tender and succulent, artful and cooked to perfection. My partner decided on the duck breast basted with a lemon honey glaze in Marsala sauce it was also a masterpiece. All main dishes are served with a full selection of vegetables in season, gratin dauphinoise, and a large mixed salad. I think all restaurants should include the vegetables in the price of the meal and I admire Popjoys for its no hidden extras.

There is an extensive but sensibly priced wine list. This includes Australian, Californian, German, Italian as well as wines from France of course. The French house wine is very reasonable and is good value. The 1984 Mercurey that I chose was produced by Michel Juillot and was fresh, fruity, as a good Mercurey should be. Served at just the correct temperature by Mark Heather, the manager, who prides himself on having a very good cellar. The veal and duck were certainly complemented by the wine.

For dessert a special English cheese, in this case a perfectly ripe Shropshire blue, is usually one of the choices, and as it was my birthday I decided to share a portion with my wife, well we had to finish off the wine didn't we?

Now came the last difficult decision. Should it be the warm treacle and almond tart with creme anglais; poached pears in a marquise of pink Champagne, or the homemade sorbet or ice cream? I was forced to choose the quenelles of dark chocolate mousse on a coffee and hazelnut sauce–simply divine. The oatmeal meringue topped with mango mousse in a strawberry couli was crying out to be eaten so my wife obliged, I also had a taste—it was gorgeous.

After the meal again one is invited to the drawing room. The fire blazing in the hearth gives a very welcoming atmosphere. Coffee, petit fours and liqueurs are now served, and the remarkable Eschaw cognac is a must to round off a truly memorable experience. The menu is changed every 2 months and booking is essential, as arrival times are planned with care to enable the very helpful staff to attend properly to you.

If you are in Bath to miss the Popjoy experience is a tragedy. I for one can't wait for my next visit.

Popjoys restaurant
Sawclose
Bath.

Jeremy. J. Mann **B.D.S.(U.Lond)**

